# Quaking regulates circular RNA production in cardiomyocytes

**DOI:** 10.1242/jcs.261120

**Published:** 2023-06-30

**Authors:** Pablo Montañés-Agudo, Ingeborg van der Made, Simona Aufiero, Anke J. Tijsen, Yigal M. Pinto, Esther E. Creemers

**Affiliations:** Department of Experimental Cardiology, Amsterdam Cardiovascular Sciences, Amsterdam UMC location University of Amsterdam, 1105AZ, Amsterdam, The Netherlands

**Keywords:** circRNAs, Alternative splicing, RNA-binding proteins, Quaking, Cardiomyocytes

## Abstract

Circular RNAs (circRNAs) are a class of non-coding RNA molecules that are gaining increasing attention for their roles in various pathophysiological processes. The RNA-binding protein quaking (QKI) has been identified as a regulator of circRNA formation. In this study, we investigate the role of QKI in the formation of circRNAs in the heart by performing RNA-sequencing on *Qki*-knockout mice. Loss of QKI resulted in the differential expression of 17% of the circRNAs in adult mouse hearts. Interestingly, the majority of the QKI-regulated circRNAs (58%) were derived from genes undergoing QKI-dependent splicing, indicating a relationship between back-splicing and linear splicing. We compared these QKI-dependent circRNAs with those regulated by RBM20, another cardiac splicing factor essential for circRNA formation. We found that QKI and RBM20 regulate the formation of a distinct, but partially overlapping set of circRNAs in the heart. Strikingly, many shared circRNAs were derived from the *Ttn* gene, and they were regulated in an opposite manner. Our findings indicate that QKI not only regulates alternative splicing in the heart but also the formation of circRNAs.

## Teaser

**Summary:** A genetic loss-of-function study in mice reveals that the splicing factor Quaking (QKI) is a crucial regulator of circular RNA formation in the heart.

## INTRODUCTION

Circular RNAs (circRNAs) are a class of non-coding RNA molecules that are increasingly being recognized for their regulatory roles in critical biological pathways. Although the function of most circRNAs is currently unknown, it has been shown that some circRNAs exert a regulatory function by acting as miRNA sponges, as protein-scaffolding molecules or as transcription or splicing regulators (Aufiero et al., 2019). Some circRNAs have even been shown to possess protein-coding potential (Legnini et al., 2017; Pamudurti et al., 2017; Yang et al., 2017). In the human heart, thousands of different circRNA molecules are expressed, with the highest numbers produced from the *TTN* and *RYR2* transcripts (Aufiero et al., 2019; Chen et al., 2016; Jakobi et al., 2020; Tan et al., 2017). Multiple circRNAs have been linked to pathological processes. For instance, *circFOXO3*, which is increasingly expressed in aging hearts, has been shown to be involved in senescence and doxorubicin-induced cardiomyopathy in the mouse heart, by interacting with anti-senescent protein ID1 and the transcription factor E2F1 (Du et al., 2017). The highly conserved *circRNA-INSR*, derived from the host gene encoding the insulin receptor, has been shown to have protective effects in doxorubicin-induced cardiomyopathy. Interestingly, *circRNA-INSR* interacts with single-stranded DNA-binding protein 1 (SSBP1) to preserve mitochondrial DNA stability and prevent cardiomyocyte apoptosis, after doxorubicin treatment in mice (Lu et al., 2022). In human induced pluripotent stem cell-derived cardiomyocytes (hiPSC-CMs), evidence has been provided that circRNAs derived from the *TTN* transcript are involved in alternative splicing by affecting the function of the splicing factors SRSF10 and RBM20 (Tijsen et al., 2021).

As our knowledge of circRNAs in the heart keeps growing, so does the need to understand the factors that control its formation. CircRNAs are generated by a non-canonical form of RNA splicing known as back-splicing, where the splice donor site of one exon is ligated to the splice acceptor site of an upstream exon. This back-splicing event creates a covalently closed single-stranded circular RNA structure consisting of one or multiple exons, and in some cases also introns. Owing to the unusual disposition of the exons within this circle, a new sequence called the back-spliced junction (BSJ) is formed. The BSJ is not present in the linear transcripts and is used to identify the circRNAs from RNA-sequencing reads. Mechanistically, the back-splicing reaction is executed by the spliceosome, and it requires that the two introns flanking the back-spliced exons are brought into close proximity of each other. This can be achieved by looping of the pre-mRNA, mediated by base-pairing of cis-acting elements (e.g. by complementary Alu repeats flanking the back-spliced exons) or by dimerization of trans-acting elements, such as splicing factors (Aufiero et al., 2019). One of the splicing factors that has been shown to regulate back-splicing is the RNA-binding protein quaking (QKI) (Conn et al., 2015).

Our laboratory has recently generated a mouse model with a conditional deletion of *Qki* in cardiomyocytes to study the function of QKI in the heart (Montañés-Agudo et al., 2023a). Removal of *Qki* in the adult heart induces dilation of the ventricles and a rapid decline in cardiac function, and this is associated with severe disruption of sarcomere organization. Numerous changes in alternative splicing were identified, mostly in sarcomeric, cytoskeletal and Ca^2+^-handling genes. We thus concluded that QKI is a major regulator of striated muscle identity by governing a muscle-specific alternative splicing program in cardiomyocytes (Montañés-Agudo et al., 2023a). This extended earlier studies, in which an essential role for QKI was demonstrated in cardiomyocyte differentiation of cardiac progenitors and in the specification of cardiac mesoderm (Chen et al., 2021; Fagg et al., 2022).

However, as a splicing factor, QKI has not only been shown to regulate alternative splicing, but also back-splicing. In an elegant study, Conn et al. (2015) developed a minigene-based reporter system to screen for RNA-binding proteins that control circRNA formation during human epithelial-to-mesenchymal transition (EMT). They showed that the production of over one-third of abundant circRNAs is dynamically regulated by QKI. Three lines of evidence subsequently revealed that QKI plays a direct role in circRNA production: (1) they found an enrichment of QKI-binding motifs in introns flanking QKI-regulated circRNAs, (2) QKI was shown to bind to sites flanking circRNA-forming exons, and (3) insertion of synthetic QKI-binding motifs into linear RNA was sufficient to induce exon circularization (Conn et al., 2015).

Given the essential role of QKI in the alternative splicing program of cardiomyocytes (Montañés-Agudo et al., 2023a), and the identification of QKI as a major regulator of circRNA formation during EMT (Conn et al., 2015), we hypothesized that QKI is also crucial for the formation of circRNAs in the heart. In this study, we explored QKI-dependent circRNA formation in the heart by using our previously characterized cardiomyocyte-specific *Qki*-knockout (KO) mouse model. By performing ultra-deep RNA-sequencing and *de novo* circRNA prediction, we show that 17% of the circRNAs that are present in the adult heart are differentially expressed after loss of QKI. We uncovered a relationship between back-splicing and linear splicing by QKI, as the majority of the QKI-regulated circRNAs (58%) were found to be derived from mRNAs that underwent QKI-dependent alternative splicing as well. Finally, we compared the differentially expressed circRNAs in the *Qki* KO model with those in an *Rbm20* KO model and found that QKI and RBM20 regulate the formation of a distinct, but overlapping, set of circRNAs. Strikingly, the *Ttn*-derived circRNAs that are regulated by QKI and by RBM20, are mostly regulated in an opposite manner.

## RESULTS

### QKI regulates formation of circRNAs in adult cardiomyocytes

To study QKI-dependent circRNA formation in the heart, we used the cardiomyocyte-specific *Qki* knockout (KO) mouse line that we previously developed. This mouse line was generated by crossing the QKI-floxed mice (Darbelli et al., 2016) with the tamoxifen-inducible Myh6-MerCreMer (*Myh6-MCM^Tg^*) line (Sohal et al., 2001). Deletion of *Qki* in adult cardiomyocytes resulted in a heart failure phenotype, consisting of an ∼45% reduction in ejection fraction and enlargement of the ventricles, which developed within 1 week after tamoxifen injections. This phenotype was accompanied by an increased expression of markers of cardiac stress, such as *Nppb*, *Myh7*, *Acta1*, *Ccn2* (encoding CTGF), *Casq2* and *Rcan1*. Cardiac dysfunction was attributed to sarcomere disorganization and splicing defects in important cardiac genes, such as *Ttn*, *Camk2d* and *Tpm1* (Montañés-Agudo et al., 2023a).

**Fig. 1.**
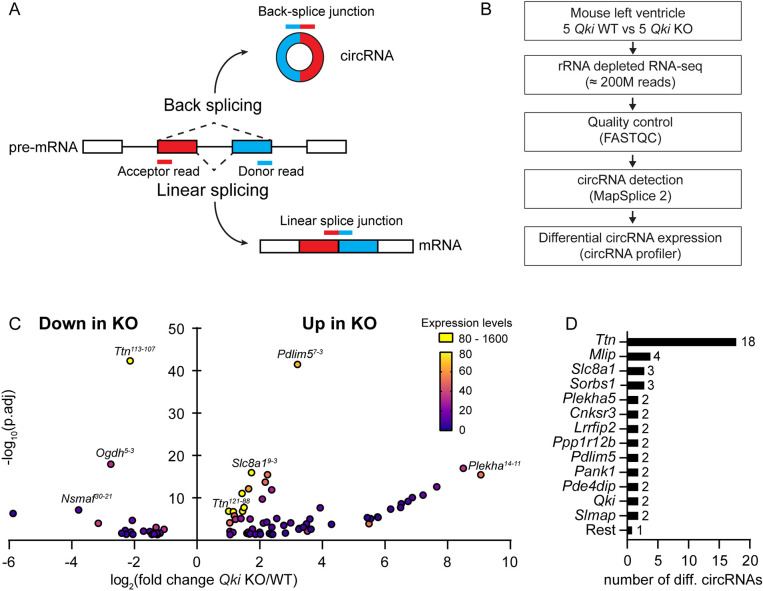
**QKI regulates circRNA production in the adult mouse heart.** (A) Diagram depicting the detection strategy to distinguish between linear splicing and back splicing. (B) Flow chart for the detection and quantification of circRNAs in *Qki* WT and KO hearts. (C) Volcano plot showing the differentially expressed circRNAs in the *Qki* KO hearts. The color code of each dot represents the expression level of each circRNA in all the samples. *n*=5 per group. (D) Number of differentially expressed circRNAs per gene in the *Qki* KO hearts. RNA-sequencing data are available at NCBI BioProject, under ID number PRJNA831665 (Montañés-Agudo et al., 2023a).

To investigate whether QKI regulates circRNA formation in cardiomyocytes, we analyzed our previous RNA-sequencing data of the *Qki* KO model for the expression of circRNAs (NCBI bioproject: PRJNA831665; Montañés-Agudo et al., 2023a). This dataset was generated from ribosomal-depleted total RNA libraries, which contained a wide variety of RNA species, including circRNAs. RNA-seq was performed on left ventricles of five conditional inducible *Qki* knockout hearts (*Qki* KO=*Qki^fl/fl^; Myh6-MCM^Tg^*) and five wild-type hearts (*Qki* WT=*Qki^wt/wt^; Myh6-MCM^Tg^*), 1 week after tamoxifen injections. CircRNA detection was performed with the bioinformatics tool MapSplice2 (Wang et al., 2010), which identifies circRNAs based on reads covering back-spliced junctions (BSJ) (Fig. 1A,B). A total of 4190 unique circRNAs were detected across the 10 samples. We filtered out the lowly and inconsistently expressed circRNAs and continued our analysis with 494 detected circRNAs that had at least three normalized read counts in all samples for the same condition. Among the most highly expressed circRNAs in the *Qki* WT heart, we found several circRNAs previously described in the mouse and human heart (Khan et al., 2016; Tan et al., 2017; Werfel et al., 2016), such as *circTtn^121-88^*, *circTtn^121-87^ and circSlc8a1^2-2^* (annotation used: *circGene^back-spliced exons^*) (see data at https://doi.org/10.6084/m9.figshare.23056598.v1).

Next, we performed differential expression analysis of circRNAs between the adult *Qki* KO and WT samples with the bioinformatics tool circRNAprofiler (Aufiero, 2020). Interestingly, 17% of the detected circRNAs were differentially expressed in the adult heart after loss of QKI [i.e. 86 of the 494 detected circRNAs, absolute log_2_(fold change) (log_2_FC) ≥1.0, adjusted *P*≤0.05], with 25 circRNAs being downregulated and 61 upregulated (Fig. 1C; Table S1). Remarkably, one circRNA was completely absent (*circSlmap^23-13^*) and 13 circRNAs were uniquely expressed in the *Qki* KO mice (arising from *Plekha5*, *Qki*, *Mlip*, *Ehbp1*, *Slmap*, *Pde4dip*, *Strn3*, *Ppp1r12b*, *Obscn*, *Cnksr3* and *Agtpbp1*). We also noted that many genes give rise to more than one dysregulated circRNA, with *Ttn* being the gene with the highest number of dysregulated circRNAs (Fig. 1D). These findings indicate that QKI is involved in the biogenesis of a large proportion of circRNAs in the adult heart.

Differential gene expression analysis was performed to compare the expression levels of circRNAs with the expression levels of their corresponding cognate host genes. *Qki* KO hearts presented 647 differentially expressed genes [absolute log_2_FC ≥1.0, adjusted *P*≤0.05, transcripts per kilobase million (TPM) ≥0.5], but there was no significant correlation with the expression of circRNAs arising from these genes. This indicates that the differences observed in circRNA formation are not dependent on changes in transcription (Fig. S1), which is in line with results from previous studies (Khan et al., 2016; Siede et al., 2017; Werfel et al., 2016).

We asked whether the large differences in circRNA expression that we observed in the *Qki* KO hearts (i.e. 17% of the expressed circRNAs) could in part be explained by more generic changes induced during the development of the cardiomyopathy. To dissect the changes that are specific for loss of *Qki*, we quantified circRNA expression in hearts where cardiac dysfunction was induced by transverse aortic constriction (TAC) as compared to sham surgery (*n*=4 sham versus 4 TAC). At 3 weeks after TAC surgery, hearts became hypertrophic and displayed a reduced ejection fraction (Fig. S2A,B). CircRNAs were detected in RNA sequencing reads using the same bioinformatics pipeline as for the *Qki* KO study. Interestingly, in this model only 3% of the circRNAs (4 out of 129 circRNAs) were differently expressed in TAC hearts compared to the sham operated control hearts (Fig. S2C). Only *circTtn^121-87^* was also changed in the *Qki* KO hearts, but the change was in the opposite direction. Interestingly, the upregulation of *circTtn^121-87^* has been previously observed in another 3-week TAC study (Werfel et al., 2016). Although the disease mechanisms after TAC and loss of *Qki* differ, these findings suggest that more general cardiac remodeling and dysfunction by itself does not lead to robust changes in circRNA expression.

### Experimental validation of the QKI-dependent circRNAs

**Fig. 2.**
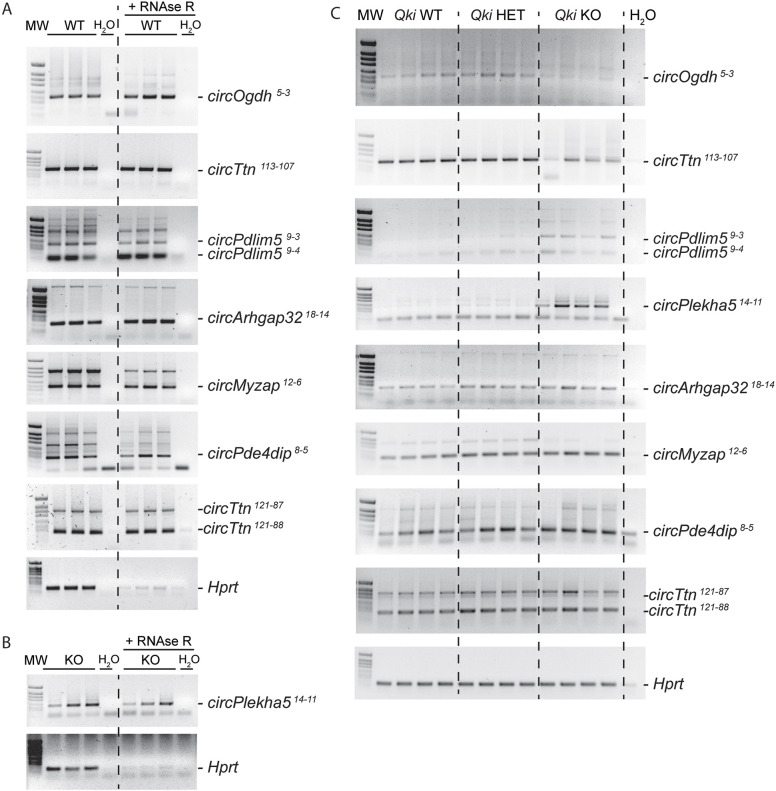
**Experimental validation of detected circRNAs.** (A) RT-PCR verification of a selection of the detected circRNAs in left ventricle samples with and without RNase R treatment of three WT and (B) three *Qki* KO mice. (C) RT-PCRs showing differentially expressed circRNAs in 4 *Qki* WT, 4 *Qki* HET and 4 *Qki* KO adult mice. Molecular ladder (MW) sizes are 712, 481, 404, 363, 242, 190, 147, 118 and 110 bp. Gels shown are from a single experiment.

To test whether the QKI-dependent circRNAs identified by RNA- sequencing, were bona fide circRNAs, we selected ten circRNAs for validation by RT-PCR. The selection of these ten circRNAs (i.e. *circOgdh^5-3^*, *circTtn^113-107^*, *circPdlim5^9-3^*, *circPdlim5^9-4^*, *circArhgap32^18-14^*, *circMyzap^12-6^*, *circPde4dip^8-5^*, *circTtn^121-87^* and *circTtn^121-88^*, *circPlekha5^14-11^*) was based on expression levels and statistical significance in the *Qki* WT versus *Qki* KO comparison. In order to exclusively amplify the circRNA molecules and not the linear transcripts, divergent primers were designed flanking the BSJ, with the forward primer annealing to the downstream exon and the reverse primer to the upstream exon. As shown in Fig. 2A, we could confirm the presence of all tested circRNAs in adult mouse hearts by RT-PCR. Of note, *circPlekha5^14-11^* was validated in *Qki* KO samples, given that this circRNA was only detected in the KO and not in the WT hearts (Fig. 2B). We also tested their expected resistance to digestion by the exoribonuclease RNase R. Whereas the linear *Hprt* transcript was clearly sensitive to RNase R digestion, the 10 circRNAs were all resistant to RNase R digestion. Finally, Sanger sequencing was performed and confirmed the presence of the BSJ in the amplicons of the ten tested circRNAs (Fig. S3). Together, these experiments demonstrate that the BSJs identified by MapSplice2 represent bona fide circRNAs.

Next, we verified the changes in circRNA expression between the *Qki* genotypes by RT-PCR (Fig. 2C). Here, we included *Qki* heterozygous (HET) hearts to check whether they presented an intermediate circRNA expression level. We confirmed the reduction in *circOgdh^5-3^* and *circTtn^113-107^* in the *Qki* KO; and the increase in *circPdlim5^9-3^*, *circPdlim5^9-4^*, *circPlekha5^14-11^*, *circArhgap32^18-14^*, *circMyzap^12-6^*, *circPde4dip^8-5^*, *circTtn^121-87^* and *circTtn^121-88^* (Fig. S4). Heterozygous mice did not show intermediate levels of expression for most of the circRNAs, except for *circMyzap^12-6^*. This is in line with the absence of a cardiac phenotype and the lack of strong splicing changes in the heterozygous KO mice (Montañés-Agudo et al., 2023a).

### Regulation of linear splicing and back-splicing by QKI

**Fig. 3.**
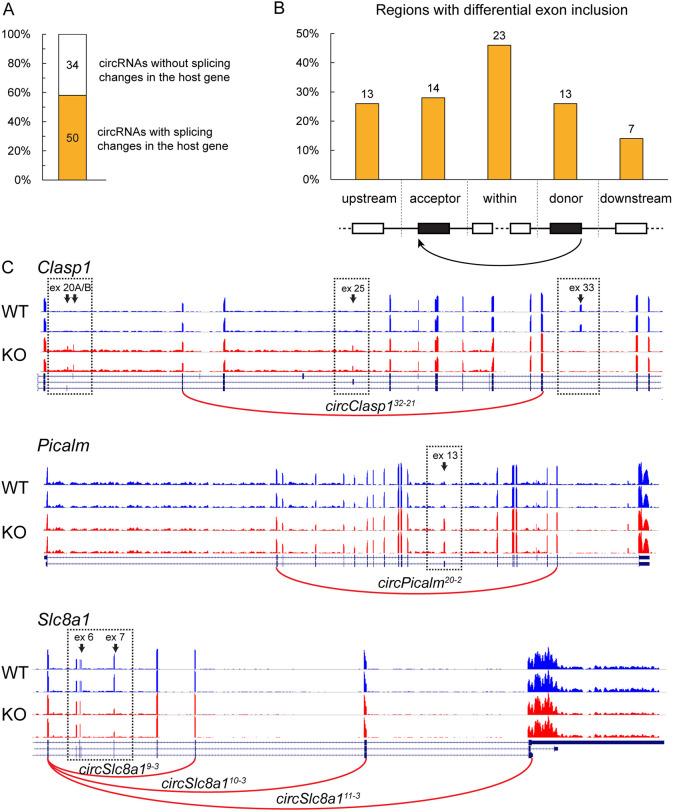
**A majority of the QKI-dependent circRNAs is linked to alternative splicing.** (A) Number of QKI-dependent circRNAs that are associated with changes in exon usage of the corresponding host gene in the *Qki* KO mice. *n*=5 per group. (B) Number (above bars) and percentages of QKI-dependent circRNAs arising from regions that also underwent alternative splicing changes in the *Qki* KO hearts. The back-spliced region was divided in five parts: acceptor and donor back-spliced exons, exons between the back-spliced exons, and exons immediately upstream and downstream of the back-spliced exons. (C) Representative examples of QKI-regulated circRNAs with differentially spliced exons (ex) in their back-spliced regions. Bed graphs show exon coverage of two mice per genotype. Black arrows indicate the significantly different exons. Red arcs indicate the significantly differently expressed circRNAs in *Qki* KO hearts.

Given that QKI activity is essential for proper splicing of multiple cardiac genes, we investigated whether changes in circRNA formation were associated with changes in alternative splicing. To do so, we compared the 86 differentially expressed circRNAs in the *Qki* KO mice with the alternatively spliced exons of the corresponding host genes as identified by DEXSeq (Montañés-Agudo et al., 2023a). We found that 50 of the 86 differently expressed circRNAs (58%) displayed at least one alternatively spliced exon in the host gene (Fig. 3A).

In order to assess where the QKI-mediated alternative splicing changes were occurring in these 50 circRNAs, we focused on the regions were circRNAs were arising and we divided it into five parts: (1) the acceptor back-spliced exon, (2) the donor back-spliced exon, (3) exons located between the back-spliced exons, and exons (4) immediately upstream and (5) immediately downstream of the back-spliced exons (Fig. 3B). We counted the number of circRNAs with alternative splicing changes (i.e. differential exon usage) in each location and found alternative splicing changes most frequently taking place in exons within the back-spliced exons (23/50) [22 of the 50 circRNAs revealed alternative splicing changes for the exons used for circularization (acceptor exon, *n*=14; donor exon; *n*=13) and 19 circRNAs were associated with splicing changes upstream and/or downstream of the back-spliced exons (upstream, *n*=13; downstream, *n*=7)]. Fig. 3C depicts three examples of genes in which alternative splicing events are associated with circRNA expression upon QKI loss (i.e. *circClasp1^32-21^*, *circPicalm^20-2^*, *circSlc8a1^9-3^*, *circSlc8a1^10-3^* and *circSlc8a1^11-^*^3^). All five circRNAs shown contain alternatively spliced exons between the back-spliced exons. *circClasp1^32^*^-21^ is an interesting circRNA, as it shows, besides alternative splicing changes in an exon located between the back-spliced exons, also alternative splicing changes in two exons immediately upstream and immediately downstream the back-spliced exons. In conclusion, our findings reveal that regions with QKI-dependent back-splicing are often associated with QKI-dependent linear splicing.

### QKI and RBM20 regulate back-splicing of a distinct, but overlapping, set of circRNAs

**Fig. 4.**
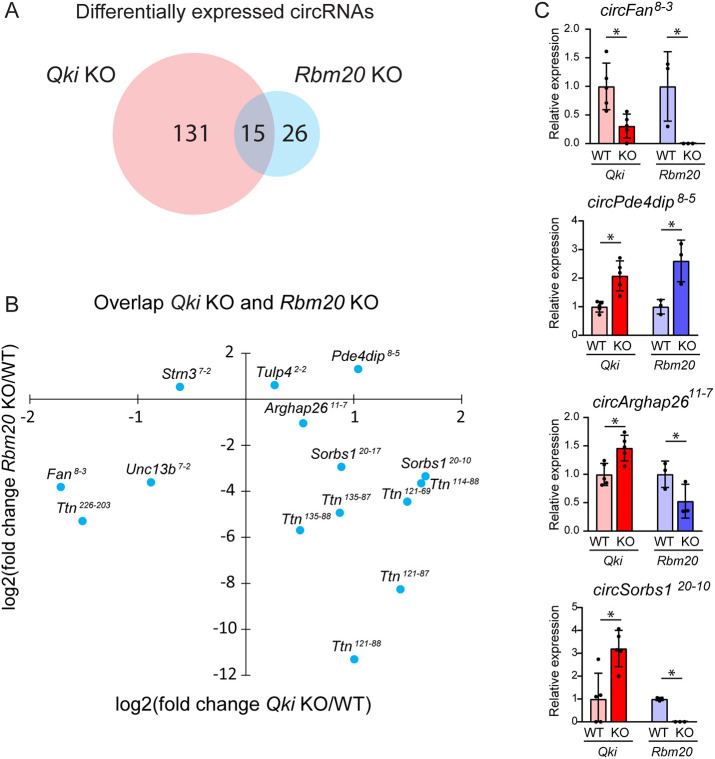
**QKI and RBM20 regulate back-splicing in distinct ways.** (A) Venn diagram showing the overlap between the differentially expressed circRNAs in the *Qki* KO and *Rbm20* KO hearts. *n*=5 per group in *Qki* model, *n*=3 per group in the *Rbm20* model. CircRNAs with absolute log_2_FC≥0.58 and *P*≤0.05 were deemed differentially expressed. (B) Scatter plot showing expression changes (log_2_FC) of the differentially expressed circRNAs in the *Qki* and *Rbm20* KO hearts. (C) Relative expression of *circFan^8-3^*, *circPde4dip^8-5^*, *circArghap26^11-7^* and *circSorbs1^20-10^* in *Qki* and *Rbm20* WT and KO mice. Relative expression is the normalized BSJ counts relative to the average of the corresponding WT. Data is presented as mean±s.d. (*n* is as for A). *P≤0.05 (statistical analysis was performed with the R Bioconductor package circRNAprofiler; Aufiero et al., 2020). RNA-sequencing data for *Qki* KO and *Rbm20* KO are available at NCBI BioProject, under ID numbers PRJNA831665 (Montañés-Agudo et al., 2023a) and PRJNA417769 (Khan et al., 2016), respectively.

Aside from QKI, there is one other splicing factor known thus far to regulate circRNA formation in the heart. This is the RNA-binding motif protein 20 (RBM20) (Khan et al., 2016; Tijsen et al., 2021), a well-studied heart and muscle-specific splicing factor mutations in which can cause inherited dilated cardiomyopathy (Guo et al., 2012). Our group has previously generated *Rbm20* KO mice to investigate the role of RBM20 in the heart, including in circRNA formation (Aufiero et al., 2018; Khan et al., 2016). These *Rbm20* KO mice manifested impaired cardiac function with dilation of the left ventricles and a 30% reduction in fractional shortening at 10 weeks of age. In these *Rbm20* KO hearts, we previously identified 41 differentially expressed circRNAs, of which 12 were produced from the *Ttn* gene (Aufiero et al., 2018). We compared the QKI-dependent and RBM20-dependent circRNAs to look for commonalities in their regulation. The Venn diagram in Fig. 4A depicts a distinct, but overlapping, set of circRNAs regulated by QKI and RBM20. A total of 15 circRNAs, arising from eight different genes, were regulated by QKI and RBM20 (absolute log_2_FC≥0.58, *P*≤0.05). The scatterplot in Fig. 4B shows the fold-changes of these 15 circRNAs in both KO models. We did not find a common direction of regulation for the overlapping circRNAs between QKI and RBM20 KO. Whereas the expression of for instance *circFan^8-3^* and *circPde4dip^8-5^* changed in the same direction in both KO models, *circSorbs1^20-10^* and *circArghap26^11-7^* expression changed in the opposite direction (Fig. 4C).

### CircRNAs derived from *Ttn* are oppositely regulated by QKI and RBM20

**Fig. 5.**
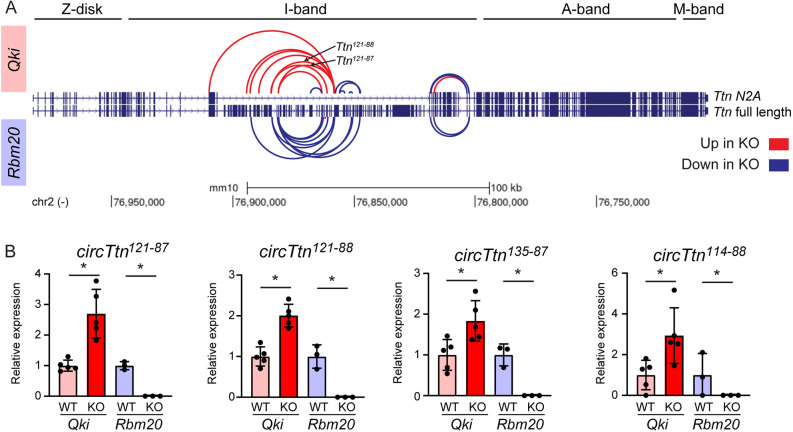
**Circular RNAs arising from *Ttn* are regulated by QKI and RBM20.** (A) Differentially expressed circRNAs arising from the *Ttn* gene in *Qki* and *Rbm20* KO hearts. *n*=5 per group in *Qki* model, *n*=3 per group in *Rbm20* model. The full-length of *Ttn* and the *Ttn* isoform N2A are shown. Each arc represents a differentially expressed circRNAs. Blue, downregulated circRNAs; red, upregulated circRNAs. (B) Expression of *circTtn* RNAs follow an opposite direction the *Qki* KO and *Rbm20* KO hearts. Relative expression is the normalized BSJ counts relative to the average of the corresponding WT. Data is presented as mean±s.d. (*n* is as for A). *P≤0.05 (statistical analysis was performed with the R Bioconductor package circRNAprofiler; Aufiero et al., 2020). RNA-sequencing data for *Qki* KO and *Rbm20* KO are available at NCBI BioProject, under ID numbers PRJNA831665 (Montañés-Agudo et al., 2023a) and PRJNA417769 (Khan et al., 2016), respectively.

Given that *Ttn* splicing has been recognized as a significant biological mechanism and that *Ttn* generates the largest quantity of circular RNAs (circRNAs), many of which exhibit differential expression in both the *Qki* KO (24 circRNAs) and the *Rbm20* KO hearts (12 circRNAs), we next explored the exact location of the back-spliced exons of the *Ttn*-derived circRNAs. First, we visualized the genomic location of the back-spliced exons of all 33 *Ttn* circRNAs detected in WT hearts (Fig. S5). As previously described (Khan et al., 2016), most *Ttn* circRNAs originate by back-splicing of exons located in the middle I-band (i.e. exons 47 to 143). This region includes the two most highly expressed circRNAs: *circTtn^121-88^* and *circTtn^121-87^*. A smaller subset of circRNAs arise from the distal I-band, but their expression levels are lower. Strikingly, when comparing the upregulated and downregulated circRNAs in the *Qki* KO and the *Rbm20* KO hearts, an opposite regulation was observed: the circRNAs in the middle I-band are increased in the *Qki* KO, but reduced (or even absent) in the *Rbm20* KO hearts (Fig. 5A,B). In conclusion, QKI and RBM20 regulate the formation of a distinct, but overlapping, set of circRNAs in an opposite manner.

## DISCUSSION

In this work, we have shown that the splicing factor QKI is crucial for circRNA formation in the heart. We found that ∼17% of the circRNAs present in the adult mouse heart are differentially spliced in the absence of QKI. Interestingly, we found circRNAs being both down- and up-regulated in the *Qki* KO, which indicates that QKI can act either as a positive or as a negative regulator of circRNA formation. We also found that changes in circRNA formation after loss of QKI are often associated with alternative splicing changes of the host transcript. These splicing changes were not only occurring in the back-spliced exons, but also between them or in close proximity.

Conn et al. were the first to postulate that QKI promotes the formation of circRNAs (Conn et al., 2015). They observed that QKI knockdown in immortalized human mammary epithelial cells led to a strong decrease in circRNA abundance. Specifically, of the 300 abundantly expressed circRNAs, 105 were decreased more than 2-fold by QKI knockdown, whereas only seven were increased (Conn et al., 2015). Similar findings have been observed in hiPSC-CMs, where inhibition of QKI by siRNAs reduced the expression of a set of five handpicked circRNAs (Lei et al., 2018). The proposed molecular mechanism by which QKI regulates circRNA formation involves QKI binding to intronic QKI-binding motifs flanking the exons to be back-spliced (Conn et al., 2015). Given that QKI exerts its function as a dimer (Beuck et al., 2012), the homodimerization of two QKI proteins surrounding these exons would bring the donor and acceptor splice sites in close proximity, thus favoring the back-splicing reaction (Conn et al., 2015). This molecular mechanism might explain the 25 downregulated circRNAs that we observed in the *Qki* KO hearts, but not the 61 upregulated circRNAs. The increased abundance of a large proportion of circRNAs in the absence of QKI in our model might point to the regulation of circRNAs in a cell-type specific manner. In this regard, it is known that the expression of RNA-binding proteins is different in different cell types. Co-expression of RNA-binding proteins might lead to competition for specific binding sites, and this could either have antagonistic or synergistic effects on splicing or circRNA formation. This, in combination with the extensive alternative splicing events occurring in the back-spliced region, point to a more complex regulation of splicing by QKI in the heart. Further research is necessary to uncover the mode of action of QKI in circRNA formation and alternative splicing. Experimentally assessing the precise location where QKI binds to the mRNA using a cross-linking immunoprecipitation assay (RNA CLIP) and identification of splicing factors that QKI interacts with across different cell types will provide insights in (back)splicing regulation by QKI in the heart.

Comparison of the *Qki* and *Rbm20* KO models revealed surprising little overlap between QKI- and RBM20-dependent circRNAs, except for circRNAs derived from the *Ttn* gene. Interestingly, *Ttn*-derived circRNAs were mostly regulated in an opposite manner by QKI and RBM20. The intricate pattern of splicing and back-splicing in the *Ttn* transcript is noteworthy for the following two reasons. First, the *Ttn* gene generates the largest number of circRNAs, which are highly expressed and mainly produced from the middle I-band region (Djebali et al., 2012; Khan et al., 2016; Werfel et al., 2016). This region is also known for extensive alternative splicing to generate diverse TTN isoforms with varying length and mechanical properties (Li et al., 2013; Loescher et al., 2022; Maatz et al., 2014). Second, multiple circRNAs from the middle I-band are regulated by QKI and RBM20 in opposite ways, with an increase in *Qki* KO and virtually complete loss in *Rbm20* KO hearts. Interestingly, alternative splicing changes in the I-band region of TTN do not point to an opposite regulation by QKI and RBM20, as I-band exons are included more in both KO models. However, there are differences. In the *Rbm20* KO hearts all exons of the I-band are included (exons 49–202), whereas in the *Qki* KO only a smaller region is included (exons 88–121) (Montañés-Agudo et al., 2023b). This smaller region controlled by QKI produces the most highly expressed circRNAs – *circTtn^121-88^* and *circTtn^121-87^*. Studies indicate that the RBM20-dependent skipped exons of the I-band are the source of circRNAs (Khan et al., 2016), as observed in *Rbm20* KO mice and in patients carrying *RBM20* mutations, where the loss of function leads to the inclusion of all exons and complete loss of circRNAs. These differences might be caused by the different ways QKI and RBM20 regulate splicing. RBM20 functions as a splicing repressor (Maatz et al., 2014), whereas QKI can act as a positive or negative regulator of splicing, depending on the position where QKI binds relative to the spliced exon (Neumann et al., 2022). The mechanism behind this behavior is not well understood, but it might involve QKI dimerizing and controlling splicing through looping of the pre-mRNA of the middle I-band (Conn et al., 2015). Adding another layer of complexity, Tijsen et al. (2021) have shown that the human *circTTN^145-79^* (which is not conserved in mouse) controls the splicing activity of RBM20 and SRSF10. This finding suggests that circRNAs derived from *Ttn* might be part of a positive-feedback loop to maintain proper splicing of *Ttn* itself.

We are not the first to study QKI-dependent circRNAs in the heart. Gupta et al. previously identified three QKI-dependent circRNAs (*circTtn^113-107^*, *circFhod3^13-11^* and *circStrn3^7-2^*) when investigating the cardioprotective function of QKI against doxorubicin toxicity (Gupta et al., 2018). In our *Qki* KO mouse model, we confirmed the QKI-dependent regulation of two of those circRNAs (i.e. *circTtn^113-107^* and *circStrn3^7-2^*), but *circFhod3^13-11^*, which was expressed in our dataset, did not appear to be a target of QKI, at least in cardiomyocytes. Some of the QKI-dependent circRNAs that we identified have been previously reported to be implicated in cardiomyocyte function. For example, decreased expression of *circTtn^113-107^* has been shown to increase doxorubicin-induced apoptosis in mouse HL-1 myocytes (Gupta et al., 2018). Although overexpressing some of these cardioprotective circRNAs could be an interesting therapeutic approach, this is complicated due to the large size of some of the circRNAs and the difficulties in promoting their circularization. An alternative strategy could be to upregulate the expression of circRNAs through modulating QKI activity. This is also interesting in light of the recent observation that cardiac QKI expression is downregulated in patients with heart failure (Gupta et al., 2018). Our previous work has already shown that QKI overexpression can enhance contractility in neonatal rat ventricular myocytes presumably by enhancing alternative splicing towards the expression of muscle-specific isoforms (Montañés-Agudo et al., 2023a). The current study suggests that changes in circRNA formation might have contributed to the increased contractile properties upon QKI overexpression as well.

Taken together, our study shows that the splicing factor QKI regulates circRNA biogenesis in adult cardiomyocytes *in vivo*. We propose that QKI is important in cardiac physiology not only by regulating alternative splicing of specific exons, but also by regulating the formation of circRNAs. Functional follow-up studies on QKI-regulated circRNAs are required to shed light on the relevance of these circRNAs in the healthy and diseased heart.

## MATERIALS AND METHODS

### Knockout mouse models

Animal studies were approved by the Institutional Animal Care and Use Committee of the University of Amsterdam and carried out in compliance with the guidelines of this institution and the Directive 2010/63/EU of the European Parliament. Animal husbandry was performed by the Animal Research Institute AMC.

Generation of the cardiomyocyte-specific *Qki* KO and inducible cardiomyocyte-specific QKI KO mice was described previously (Montañés-Agudo et al., 2023a). In brief, mice were generated by crossing the *Qki*-floxed line (Darbelli et al., 2016) with the tamoxifen-inducible Myh6-MerCreMer (*Myh6-MCM*) (Jackson Laboratory stock #005657) (Sohal et al., 2001) line in the C57BL/6N background. For inducing MCM recombinase activity, a tamoxifen solution (2.5 mg/ml tamoxifen in 10% ethanol, 90% sunflower oil) was injected intraperitoneally for 4 consecutive days (total dose 100 mg tamoxifen/kg body weight mouse) in 12–17-week-old mice (*n* per group: 5 *Qki* WT, 6 *Qki* HET, 9 *Qki* KO). The MCM recombinase allele was present in one copy in all the mice. Both males and females were included in the experiments. *Rbm20* KO mice were previously generated and characterized (Khan et al., 2016).

### Transverse aorta constriction

Transverse aorta constriction (TAC) was performed in 8-week-old male mice [mixed background (F2) of C57BL/6 and FVB] as previously described (Tijsen et al., 2014). At 3 weeks after TAC or sham surgery, echocardiography was performed as previously described (Montañés-Agudo et al., 2023a) and mice were killed while being sedated.

### RNA sequencing

RNA sequencing of the Qki KO mice used in this study had been previously performed [NCBI bioprojects PRJNA831665 (*Qki* KO); Montañés-Agudo et al., 2023a]. In short, total RNA from left ventricle tissue of adult mice (5 WT and 5 *Qki* KO) was extracted by using TRI reagent (Sigma, Ref. T9424). RNA quality was determined using the Agilent RNA 6000 Nano Kit and the Agilent 2100 Bioanalyser. All samples had an RNA Integrity score ≥8. Library preparations were made with the Kapa RNA Hyperprep with RiboErase (Roche) and sequenced on a NovaSeq platform (NovaSeq S4.300; flow cell type PE150, 2×150nt, sequencing depth ∼108.5–225.5 million reads per sample). Quality control of FASTQ files was performed using FASTQC (Babraham Bioinformatics - FastQC A Quality Control tool for High Throughput Sequence Data). Trimmomatic (version 0.351) (Bolger et al., 2014) was used to remove adapters and low-quality bases, using a Phred score cutoff of 30, while discarding reads with a length below 75 bases. The paired-end RNA-seq reads passing the quality controls from the five *Qki* KO mice and five wild-type mice were then aligned against the mouse genome, Gencode annotation release vM25 (GRCm38/mm10), using MapSplice2 (version 2.2.0). For circRNAs detection, we set the following options: --min-fusion-distance 200 (as suggested by the authors), --filtering 1, and --min-map-len 50.

Differential gene expression analysis was performed using the R Bioconductor package, DESeq2 (Love et al., 2014) (Bioconductor release 3.13). Transcripts per million (TPM) for each gene were also calculated. Genes with TPM value ≥0.5, an absolute log_2_FC≥1.0 and adjusted *P*≤0.05 were deemed significantly differentially expressed (Montañés-Agudo et al., 2023a).

Differential exon usage analysis was performed using the R Bioconductor package, DEXSeq (Anders et al., 2012) (Bioconductor release 3.13). Only genes expressed with TPM value ≥0.5 were considered. Exon bins with absolute log_2_FC≥1.0 and adjusted *P*≤0.05 were deemed differentially spliced (Montañés-Agudo et al., 2023a).

Differential circRNA expression analysis was performed using the R Bioconductor package circRNAprofiler (Aufiero, 2020) (Bioconductor release 3.13). The results from this analysis are available in the Figshare repository at https://doi.org/10.6084/m9.figshare.23056598.v1. CircRNAs with an absolute log_2_FC≥1.0 and adjusted *P*≤0.05 were deemed significantly differentially expressed in the *Qki* KO hearts (Table S1).

RNA sequencing of the 3-week TAC mice (4 sham, 4 TAC) was performed at QIAGEN Benelux B.V. (The Netherlands) (NextSeq platform 2×100 bp, sequencing depth 88–113 million reads per sample). Bioinformatic analysis was performed using the same bioinformatics pipeline as for the *Qki* KO mice.

RNA sequencing of the *Rbm20* KO mice (three WT and three KO mice) had been previously performed by Khan et al. [NCBI bioprojects PRJNA417769 (*Rbm20* KO); Khan et al., 2016]. Bioinformatic analysis was performed using the same pipeline as in the *Qki* QKI KO mice, but in the mouse genome annotation GRCm39/mm39. In order to compare the differentially expressed circRNAs in the *Rbm20* KO with the QKI-dependent circRNAs, coordinates of the back-spliced exons were converted to the GRCm38/mm10 annotation with the LiftOver tool from the UCSC genome browser (Kent et al., 2002). For comparison of QKI-dependent and RBM20-dependent circRNAs, we used the following cut-offs: log_2_FC ≥0.58 and *P*-value ≤0.05.

### RNase R digestion and RT-PCRs

To validate circularity of circRNAs, an RNase R digestion step was performed on three QKI WT and three *Qki* KO samples. Specifically, 2.5 μg of RNA were incubated in 1× RNase R buffer with or without 5 units of RNase R (Epicentre) at 37°C for 10 min followed by heat inactivation at 95°C for 3 min.

For cDNA synthesis, 500 ng of total RNA were treated with DNase I (Invitrogen, Ref 18068-015, Waltham, MA, USA) and retrotranscribed into cDNA with random hexamers (Invitrogen, Ref N8080127) and Superscript II (Invitrogen, Ref 18064-014).

RT-PCRs were performed in a 25 µl reaction containing 5 ng cDNA, 1 M Betaine, 1× Buffer B2, 2.5 mM MgCl2, 200 µM dNTPs, 0.4 µM forward primer, 0.4 µM reverse primer and 0.05 U/µ HOT FIREpol^®^ DNA polymerase (Solis Biodyne). The thermal cycling protocol included an initial denaturation at 95°C for 15 min to activate the HOT FIREPol^®^ DNA polymerase, followed by 25–35 amplification cycles of denaturation at 95°C for 30 s, annealing at 59–62°C for 30 s, extension at 72°C for 45 s, and 5 min of final extension at 72°C. Primer sequences are found in Table S2.

### Visualization of circRNAs

circRNAs were visualized on the UCSC genome browser (Kent et al., 2002) by creating a custom track with the back-spliced exons. CircRNAs were drawn manually.

### Statistics

Data is presented as mean±s.d., unless otherwise stated. Results were analyzed with statistical tests in GraphPad Prism 9 (GraphPad, San Diego, CA, USA), as indicated in the respective figure legends. A value of *P*≤0.05 was considered statistically significant. Statistical tests concerning RNA-seq data are indicated in the RNA sequencing section.

## Supplementary Material

10.1242/joces.261120_sup1 Supplementary information
