# Supplementary material for: Quaking regulates circular RNA production in cardiomyocytes
Source: J Cell Sci. 2023 Jun 30;136(13):jcs261120. doi: 10.1242/jcs.261120 (PMC10323251; doi:10.1242/jcs.261120)
Supplement: Supplementary information [file joces-136-261120-s1.pdf]

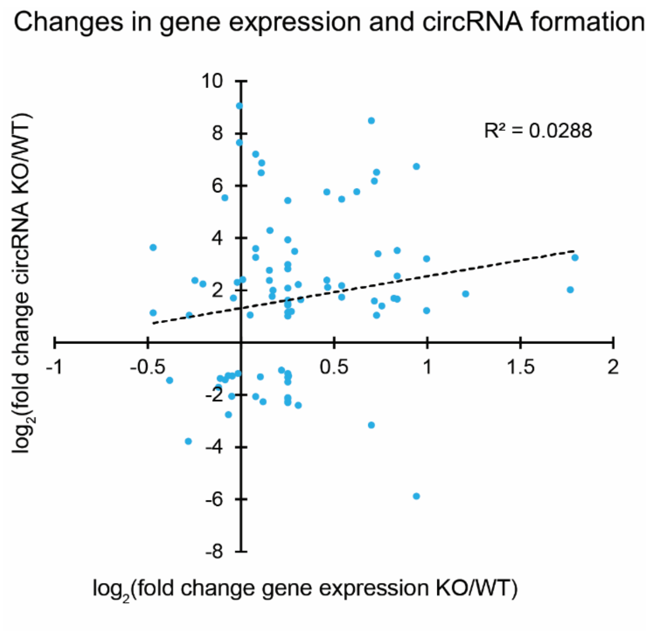

Fig. S1. There is no correlation between gene expression and circRNA formation. Scatter plot depicting the changes in circRNA expression against the changes in expression of the host gene in the *Qki* KO mice. Each dot is a circRNA. Only circRNAs with adj. p-val ≤ 0.05 are shown. n = 5 per group.

Journal of Cell Science • Supplementary information

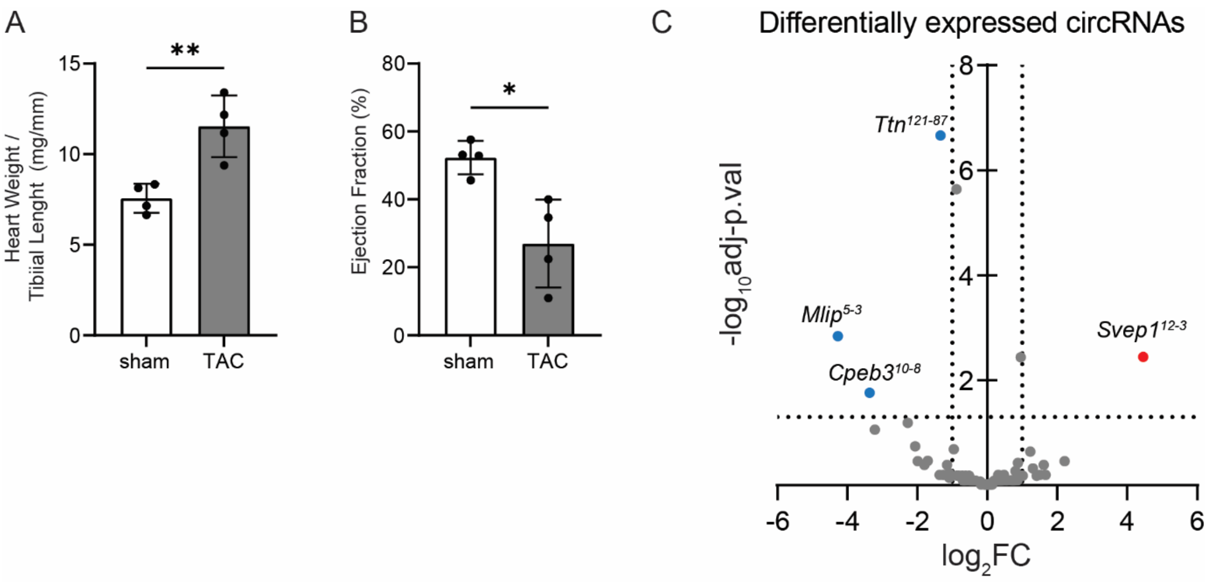

Fig. S2. Differentially expressed circRNAs in the mouse heart 3 weeks after transverse aortic constriction (TAC). (A) Normalized heart weight. (B) Ejection fraction. Data are presented as mean ± standard deviation. Unpaired t-test, * $p \leq 0.05$, ** $p \leq 0.01$. (C) Volcano plot depicting differentially circRNA expression. n = 4 per group.

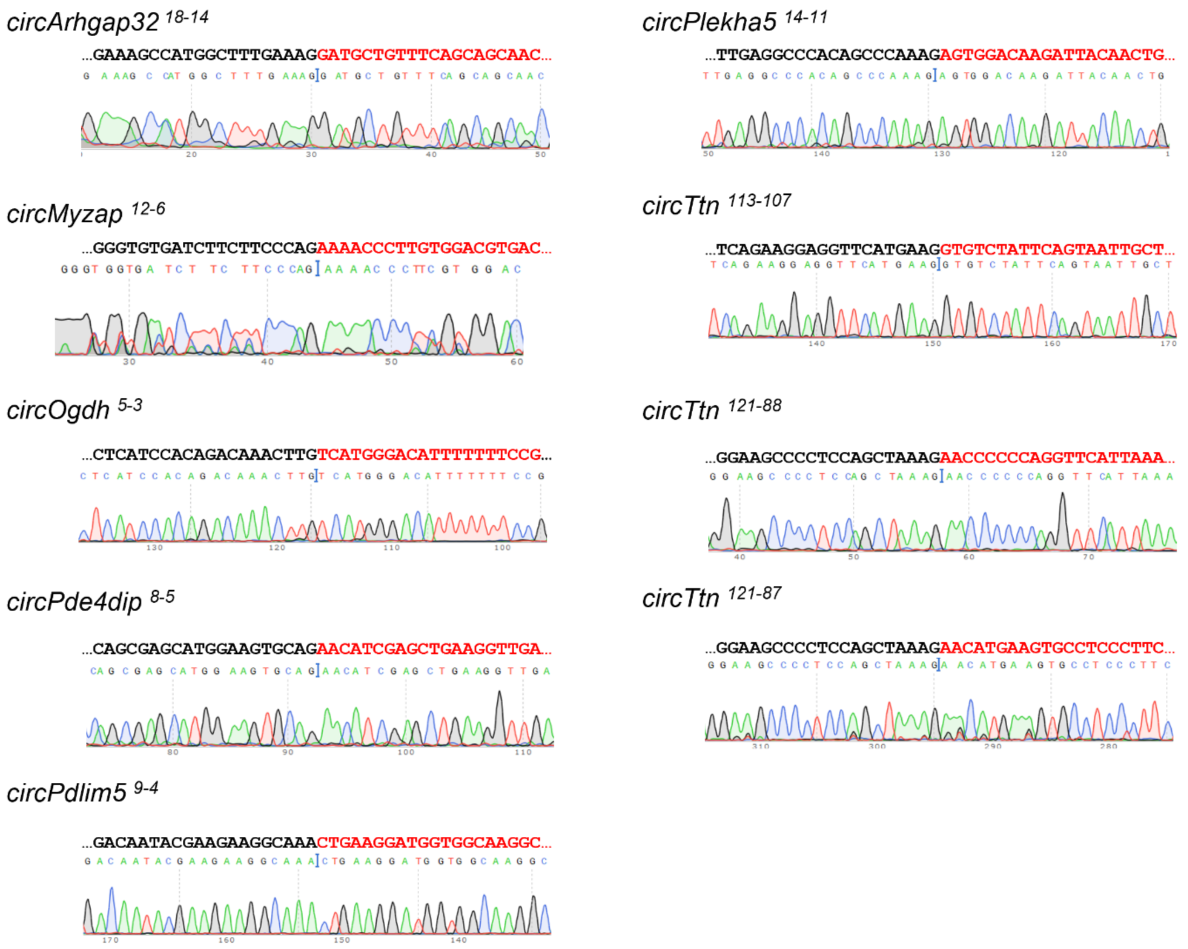

Fig. S3. Sanger sequencing verification of selected circRNAs. For each circRNA, the expected back-spliced junction sequence is indicated with the corresponding donor exon in black and acceptor in red. Chromatogram with the sequencing results is shown below.

Journal of Cell Science • Supplementary information

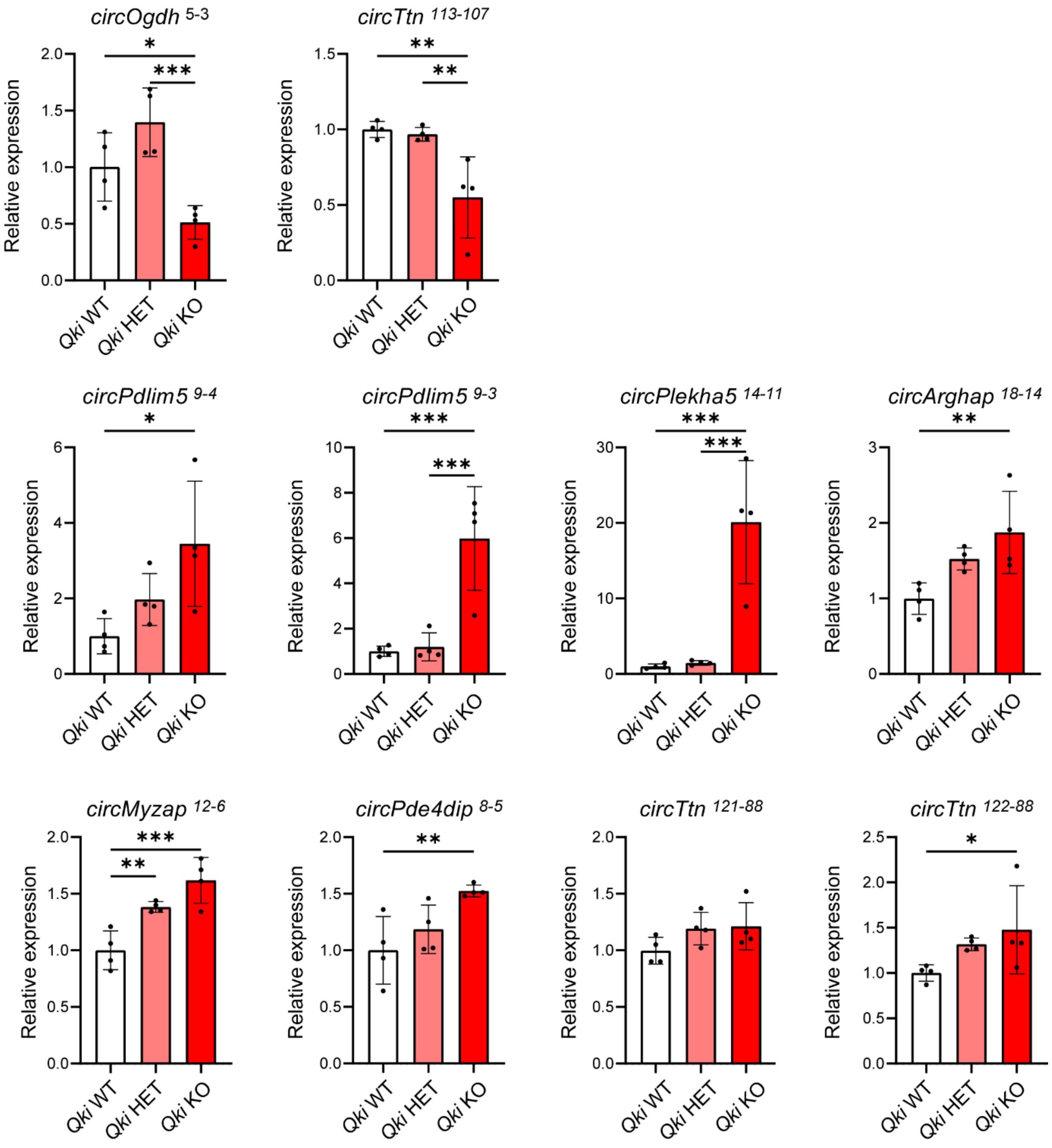

Fig. S4. Quantification of RT-PCRs shown in Figure 3C. Relative expression is the ratio between the intensity of the amplicon band of the circRNA against *Hprt*. Data are presented as mean ± standard deviation. Ordinary one-way ANOVA, followed by Uncorrected Fisher's Least Significant Difference test; **** $p \leq 0.0001$; *** $p \leq 0.001$; ** $p \leq 0.01$; * $p \leq 0.05$. n = 4 per group.

Journal of Cell Science • Supplementary information

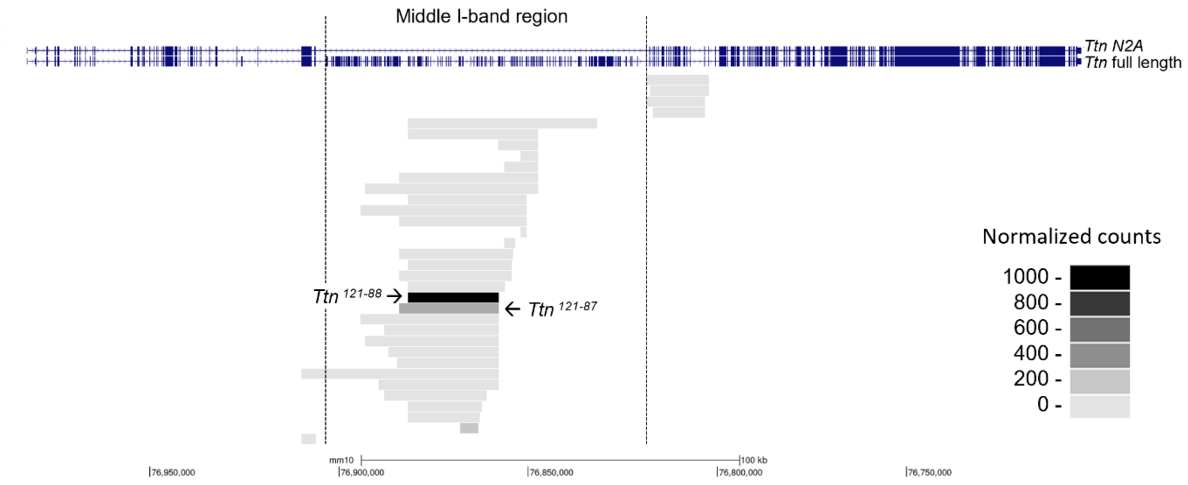

Fig. S5. Most circular RNAs arising from *Ttn* are generated from exons within the middle I-band region. View of the *Ttn* gene (full length isoform and N2A isoform) and all the *circTtn*'s detected in *Qki* WT hearts. Each bar connects the back spliced exons of each circRNA. Grey scale represents the level of expression of each circRNA (normalized counts of 5 *Qki* WT hearts).

Journal of Cell Science • Supplementary information

Table S1. List of differentially expressed circRNAs in adult *Qki* knock-out hearts

Gene	circRNA id (mm10)	Average normalized counts *Qki* WT	*Qki* KO	log2FC	adj p-val
Slmap	chr14:26428316-26416203	9,8	0,0	-5,9	5,0E-07
Nsmaf	chr4:6417207-6398319	22,7	1,6	-3,8	7,4E-08
Qki	chr17:10282981-10238893	67,5	7,6	-3,2	1,0E-04
Ogdh	chr11:6313788-6317080	57,7	8,5	-2,8	1,2E-18
St6galnac3	chr3:153411871-153411462	8,4	1,5	-2,4	2,2E-02
Ttn	chr2:76856366-76847388	14,1	2,7	-2,3	5,0E-03
Ttn	chr2:76856366-76853605	78,9	16,4	-2,3	3,5E-02
Arl8b	chr6:108813627-108818613	10,7	2,3	-2,3	4,1E-02
Ttn	chr2:76868085-76863274	201,4	45,7	-2,1	4,9E-43
Ttn	chr2:76852137-76850570	21,9	4,9	-2,1	1,1E-02
Mlip	chr9:77217048-77173959	25,1	6,0	-2,1	2,1E-05
Lamp2	chrX:38442140-38424267	9,3	2,1	-2,1	4,2E-02
Fan1	chr7:64371659-64361940	20,8	6,4	-1,7	7,4E-03
Ttn	chr2:76818816-76802231	18,9	6,8	-1,5	9,2E-03
Vav3	chr3:109562743-109578364	17,1	6,4	-1,4	4,4E-02
Nsd3	chr8:25640577-25649703	21,2	7,8	-1,4	1,3E-02
S100pbp	chr4:129183717-129178241	15,2	5,7	-1,4	3,5E-02
Ttn	chr2:76818816-76803293	18,3	7,2	-1,3	2,1E-02
Arhgap21	chr2:20860036-20855354	47,3	18,6	-1,3	9,8E-04
Ube3a	chr7:59240967-59247217	15,9	6,4	-1,3	2,2E-02
Tent4a	chr13:69515903-69512890	20,4	8,6	-1,3	2,2E-02
Psd3	chr8:67908889-67882981	19,0	8,0	-1,3	4,9E-02
Amotl1	chr9:14575439-14571623	21,3	9,7	-1,2	2,1E-02
Ttn	chr2:76857987-76847388	37,7	16,4	-1,2	2,2E-02
Spopl	chr2:23545579-23537328	44,2	21,4	-1,1	2,7E-03
Ttn	chr2:76881839-76857904	1063,3	2134,1	1,0	1,5E-07
Pde4dip	chr3:97796808-97792739	32,5	67,6	1,0	7,9E-05
Oxr1	chr15:41797474-41826052	13,3	27,1	1,0	8,3E-03
Map4k4	chr1:40003757-40010677	10,0	20,9	1,1	4,8E-02
Pank1	chr19:34841109-34812278	12,1	26,4	1,1	1,5E-02
Ttn	chr2:76888087-76857904	50,3	113,5	1,2	1,9E-07
Picalm	chr7:90160448-90197020	27,6	64,6	1,2	1,8E-06
Pdlim5	chr3:142314448-142303943	19,1	44,7	1,2	1,2E-05
Corin	chr5:72454506-72422025	14,1	35,7	1,4	8,0E-06
Ttn	chr2:76884197-76857904	342,8	925,4	1,4	9,4E-12
Ttn	chr2:76894423-76857904	56,0	154,7	1,4	1,5E-07
Ttn	chr2:76893177-76857904	43,3	122,0	1,5	1,9E-08
Ppp1r12b	chr1:134842733-134834435	8,7	25,5	1,6	2,4E-02
Ttn	chr2:76881839-76862800	8,3	24,5	1,6	1,3E-02
Arhgap32	chr9:32246447-32250787	29,2	92,6	1,6	7,6E-13
Sorbs1	chr19:40365166-40336986	5,2	16,8	1,7	3,5E-02
Slc35f5	chr1:125568547-125579327	11,4	35,3	1,7	1,0E-05
Rere	chr4:150500003-150510038	4,5	15,0	1,7	3,0E-02
Slc8a1	chr17:81445593-81428182	47,5	155,7	1,7	1,1E-16
Akap6	chr12:53139381-53142768	7,2	26,4	1,8	2,4E-03
Alpk2	chr18:65350708-65349028	3,7	12,2	1,9	4,1E-02
Zfp644	chr5:106638635-106635610	4,3	15,8	2,0	1,3E-02
Enah	chr1:181961919-181905469	2,8	12,2	2,0	4,1E-02
Ttn	chr2:76884633-76857904	9,8	41,0	2,1	2,0E-10
Asph	chr4:9639347-9630773	5,8	24,6	2,1	9,4E-05
Slc8a1	chr17:81445593-81408067	18,1	81,0	2,2	2,0E-14
Myo9a	chr9:59801914-59843138	4,3	21,1	2,2	5,4E-05
Agl	chr3:116782643-116778619	18,6	85,1	2,2	3,5E-16
Erc1	chr6:119825819-119824386	2,0	8,7	2,3	2,6E-02
Rbm41	chrX:139968599-139954907	1,0	5,9	2,4	4,2E-02

Lrrfip2	chr9:111188762-111205847	9,4	47,8	2,4	1,3E-12
Cnksr3	chr10:7154485-7152864	3,2	17,4	2,4	9,0E-04
Fkbp15	chr4:62336547-62329348	0,8	5,2	2,4	4,1E-02
Sorbs1	chr19:40377016-40340685	3,4	22,3	2,5	7,8E-06
Lrrfip2	chr9:111182819-111205847	2,6	18,0	2,8	3,1E-04
Ttn	chr2:76888087-76861097	2,0	11,0	2,8	2,1E-02
Ttn	chr2:76817075-76803293	3,1	23,2	3,0	8,1E-05
Pdlim5	chr3:142352885-142303943	13,3	121,0	3,2	3,5E-42
Mapk4	chr18:73971276-73969891	1,2	12,8	3,2	2,6E-03
Mlip	chr9:77243727-77229485	1,2	13,0	3,3	1,7E-03
Raph1	chr1:60527489-60518980	1,0	12,0	3,4	8,9E-04
Mllt10	chr2:18101458-18146872	1,5	18,9	3,5	1,4E-04
Sorbs1	chr19:40344439-40321793	4,8	56,4	3,5	7,4E-03
Mlip	chr9:77230955-77229485	1,4	17,3	3,6	1,0E-05
Pank1	chr19:34827373-34812278	0,7	9,7	3,6	2,6E-03
Ttn	chr2:76909953-76857904	1,4	23,9	3,9	2,3E-08
Clasp1	chr1:118521827-118552156	0,5	11,7	4,3	1,8E-04
Ttn	chr2:76889591-76857904	0,0	8,2	5,4	4,8E-06
Slc8a1	chr17:81445593-81388810	2,1	98,6	5,5	1,3E-04
Agtpbp1	chr13:59482604-59475660	0,0	9,0	5,5	8,3E-06
Cnksr3	chr10:7154485-7134522	0,0	10,5	5,8	3,3E-06
Obscn	chr11:59016373-59015436	0,0	10,5	5,8	4,2E-06
Ppp1r12b	chr1:134865960-134834435	0,0	13,9	6,2	4,7E-08
Strn3	chr12:51661713-51643104	0,0	17,3	6,5	1,9E-08
Pde4dip	chr3:97710444-97706872	0,0	17,6	6,5	2,5E-09
Slmap	chr14:26428316-26422421	0,0	20,4	6,7	7,0E-10
Ehbp1	chr11:22151892-22137838	0,0	22,5	6,9	9,0E-11
Mlip	chr9:77231029-77229485	0,0	28,6	7,2	2,3E-11
Plekha5	chr6:140543720-140568881	0,0	38,7	7,6	2,6E-13
Qki	chr17:10274063-10238893	0,0	69,6	8,5	1,1E-17
Plekha5	chr6:140543720-140556070	0,0	102,4	9,1	3,8E-16

Journal of Cell Science • Supplementary information

Table S2. Primer sequences

Primer	Sequence 5' - 3'	Annealing on exon
circArhgap32 Rv	GAGTTGCTGGATGACATCATGG	14
circArhgap32 Fw	CGCAAGTTGCAGCGTAATGAAAG	18
circMyzap Rv	TCTAACTGCCTCTGCTTCTCCCG	6
circMyzap Fw	GACAGAAACCCAGCCCAAGACTG	12
circNsmaf Rv	TCAAGTCTACACCACCTTCATAGGTCAG	20
circNsmaf Fw	AAGTCGGTCTGGTGAGCTGC	31
circOgdh Rv	TCGACGTTAGGTTGTGCTTCCACC	3
circOgdh Fw	TGATCTGGACTCCTCCGTGCC	5
circPde4dip Rv	TGTCCTGGAGTTCTCGTTTCAGGC	5
circPde4dip Fw	CTGAGGCAGAGCTTGGCTGC	8
circPdlim5 Rv	CACTCTTGGCTGCAGCTGAA	4
circPdlim5 Fw	GAATCTGAAAATGACAATACGAAGAAGGC	9
circPlekha5 Rv	CCGTATCTTTCAGCTTCTAAGGC	11
circPlekha5 Fw	ACACCTTAGCACAGCTCATG	14
circSorbs1 Rv	CGGTCTCCCAAACTCCAATTCCG	16
circSorbs1 Fw	CCAGACATTACGTCAGAGCCTCCTG	20
circSorbs1 Rv	TGTAGACACATCGCTCAGGTCCTG	8
circSorbs1 Fw	CCAGACATTACGTCAGAGCCTCCTG	20
circTtn Rv	TGGTTGTTAAGTACAGTTCCGCTG	107
circTtn Fw	GACTATGAAGAGATCAAGGTGGAAGC	113
circTtn Fw	CAGAGGCTCCAAAGAAACCTGCTC	121
circTtn Rv	CTGCTTCACGATCCGTGATTGGTC	88
Hprt Fw	CCTAAGATGAGCGCAAGTTGAA	9
Hprt Rv	CCACAGGACTAGAACACCTGCTAA	9
Qki Fw	GGAGTGCAGAATTGCCTG	2
Qki Rv	CTAGGTCCAAGGATTCTCC	3

Journal of Cell Science • Supplementary information
